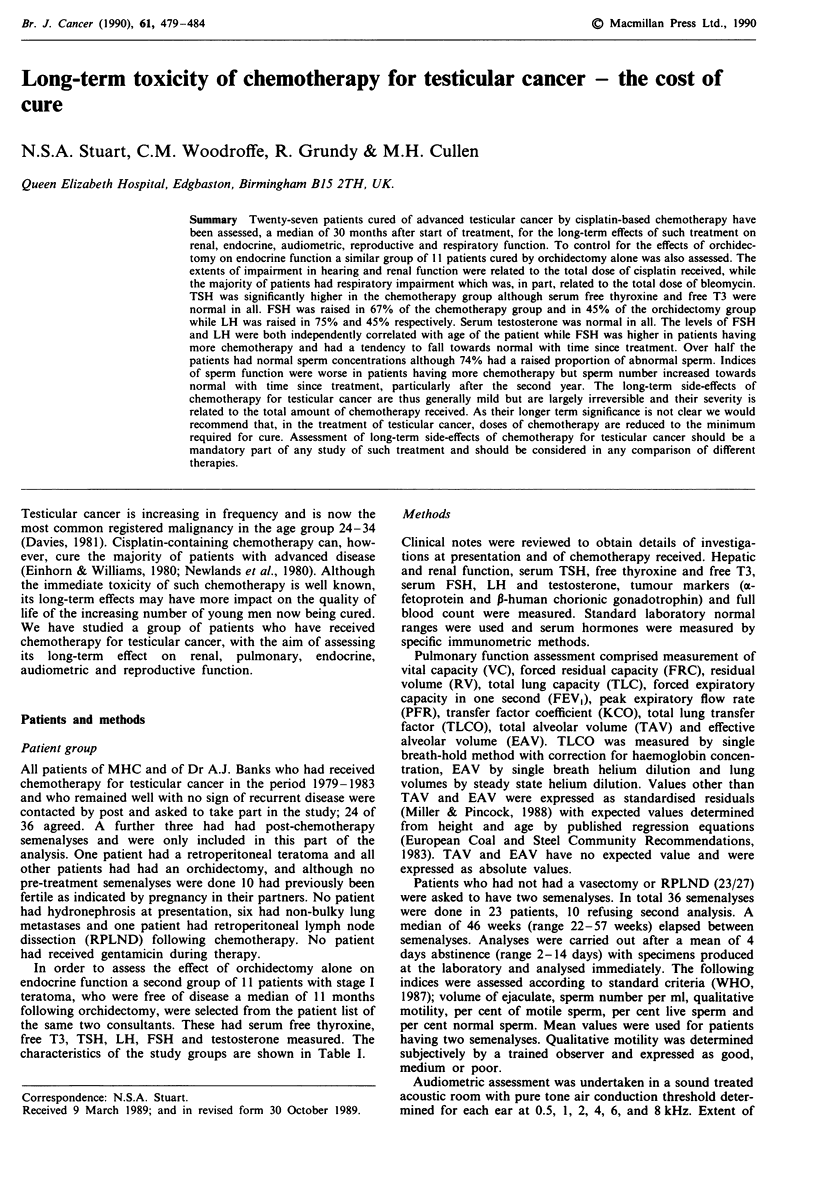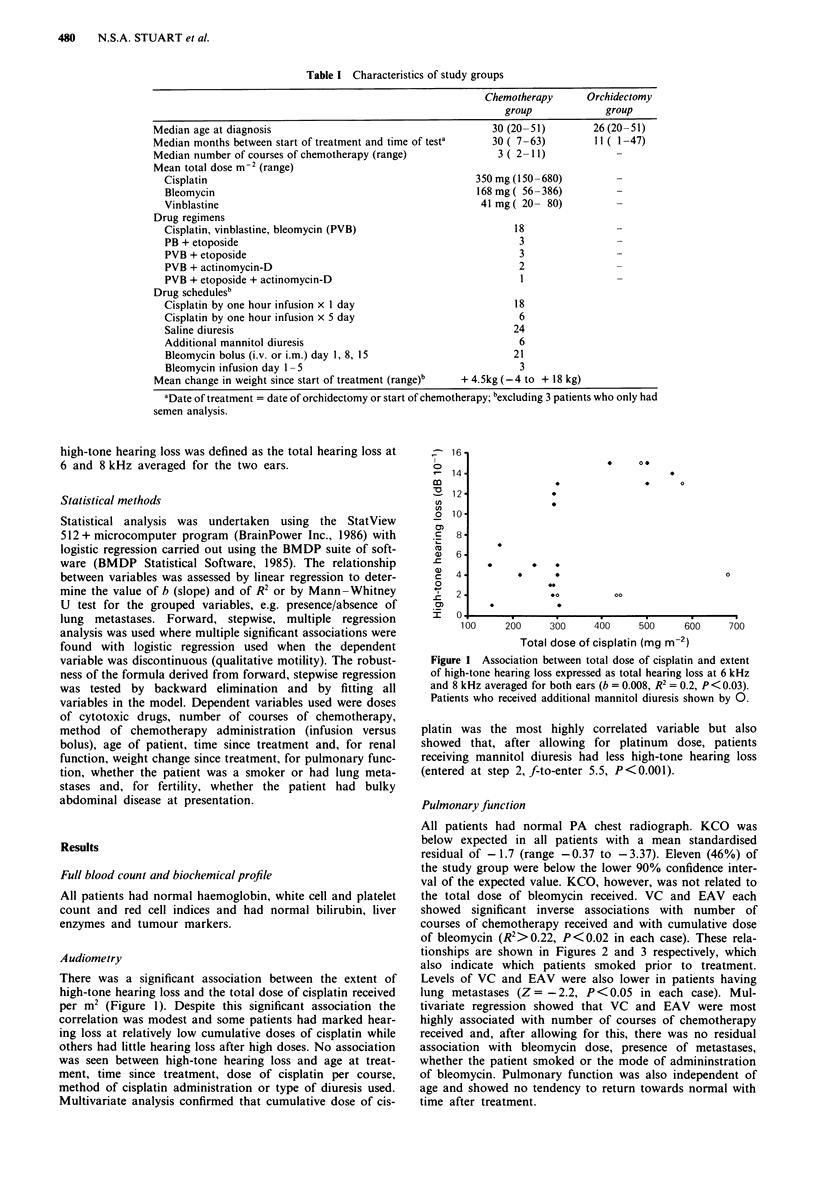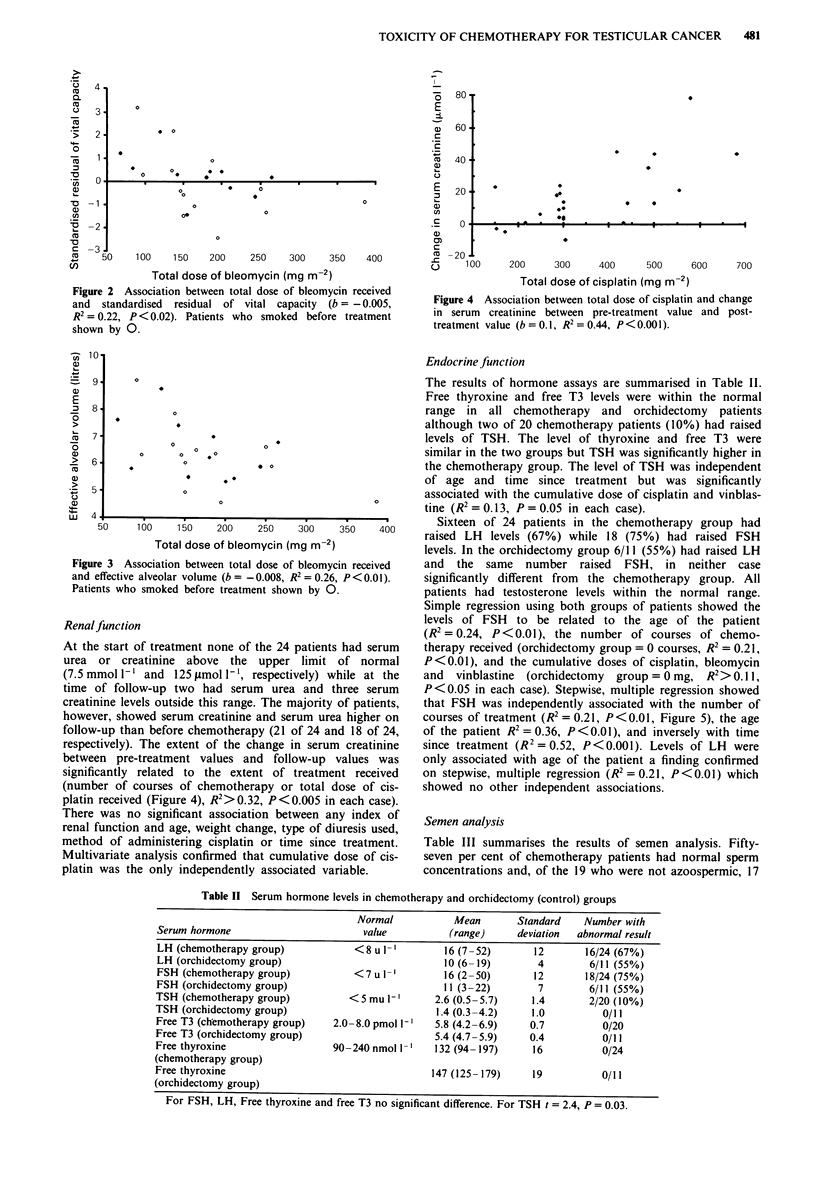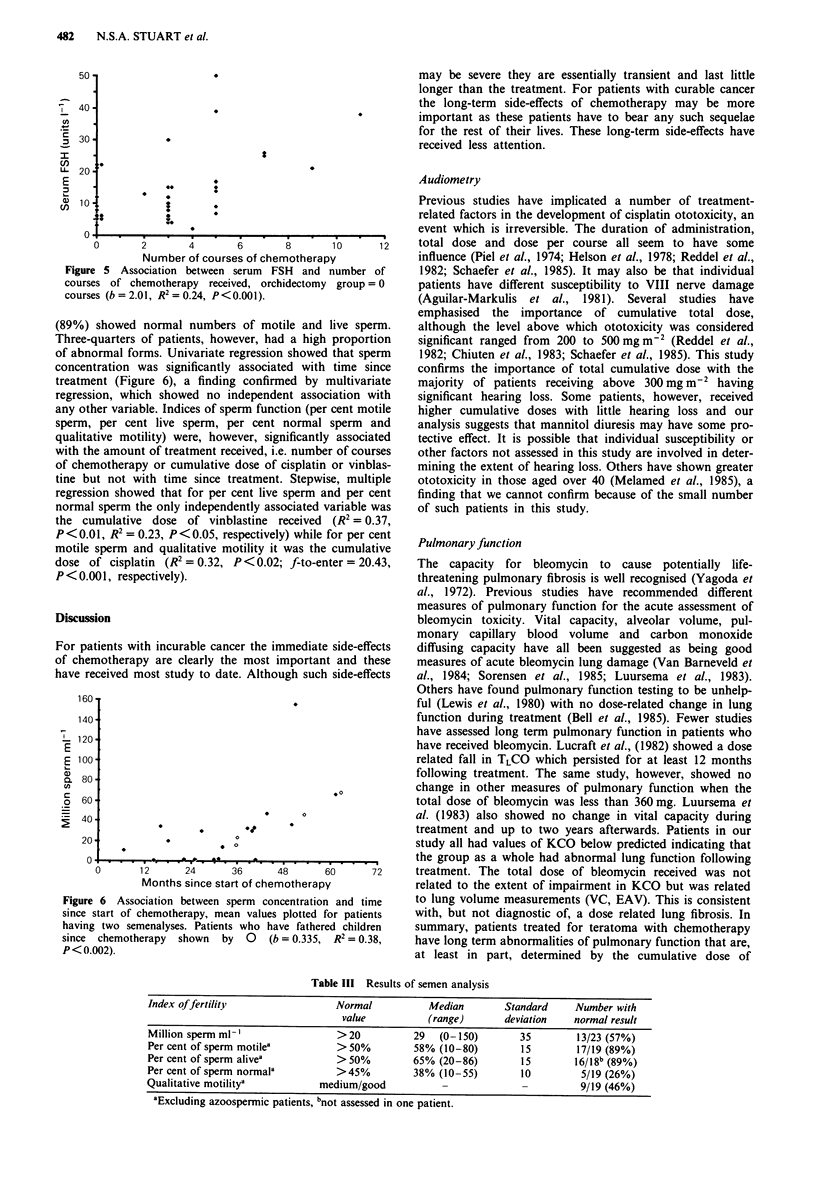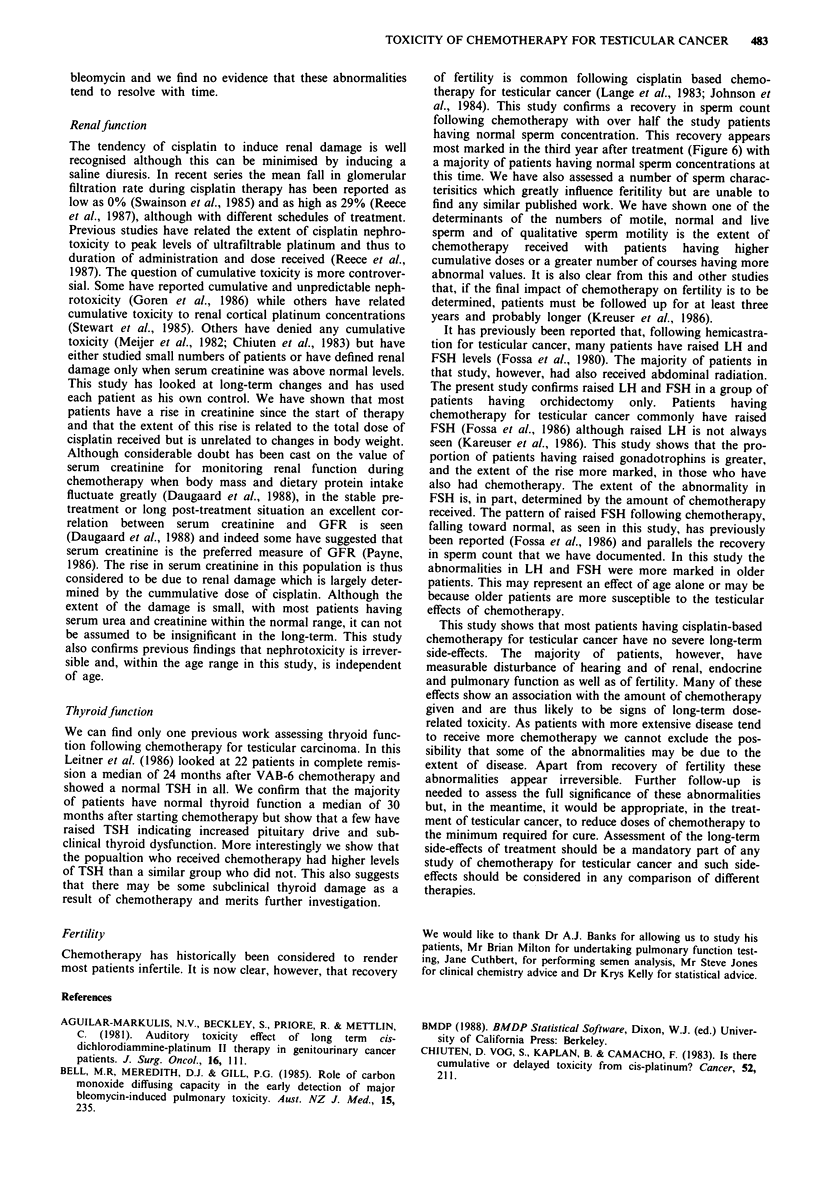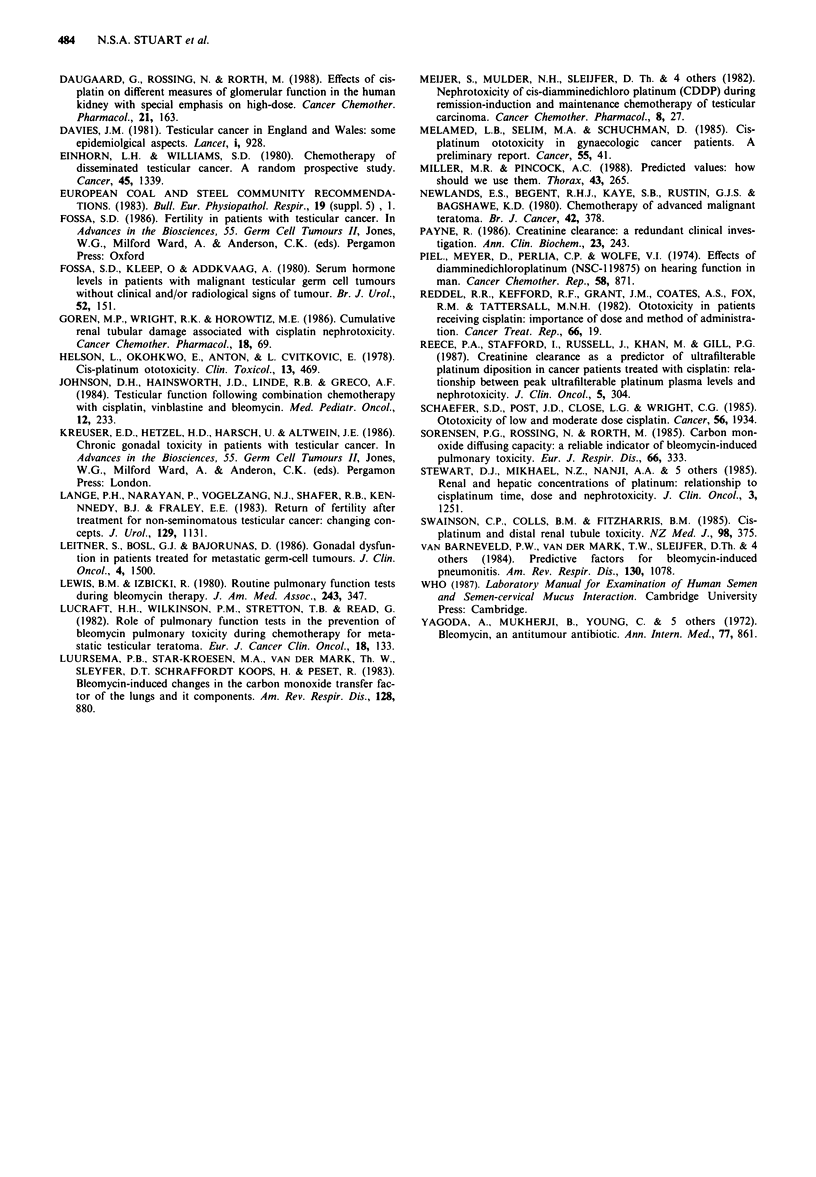# Long-term toxicity of chemotherapy for testicular cancer--the cost of cure.

**DOI:** 10.1038/bjc.1990.106

**Published:** 1990-03

**Authors:** N. S. Stuart, C. M. Woodroffe, R. Grundy, M. H. Cullen

**Affiliations:** Queen Elizabeth Hospital, Edgbaston, Birmingham, UK.

## Abstract

Twenty-seven patients cured of advanced testicular cancer by cisplatin-based chemotherapy have been assessed, a median of 30 months after start of treatment, for the long-term effects of such treatment on renal, endocrine, audiometric, reproductive and respiratory function. To control for the effects of orchidectomy on endocrine function a similar group of 11 patients cured by orchidectomy alone was also assessed. The extents of impairment in hearing and renal function were related to the total dose of cisplatin received, while the majority of patients had respiratory impairment which was, in part, related to the total dose of bleomycin. TSH was significantly higher in the chemotherapy group although serum free thyroxine and free T3 were normal in all. FSH was raised in 67% of the chemotherapy group although serum free thyroxine and free T3 were while LH was raised in 75% and 45% respectively. Serum testosterone was normal in all. The levels of FSH and LH were both independently correlated with age of the patient while FSH was higher in patients having more chemotherapy and had a tendency to fall towards normal with time since treatment. Over half the patients had normal sperm concentrations although 74% had a raised proportion of abnormal sperm. Indices of sperm function were worse in patients having more chemotherapy but sperm number increased towards normal with time since treatment, particularly after the second year. The long-term side-effects of chemotherapy for testicular cancer are thus generally mild but are largely irreversible and their severity is related to the total amount of chemotherapy received. As their longer term significance is not clear we would recommend that, in the treatment of testicular cancer, doses of chemotherapy are reduced to the minimum required for cure. Assessment of long-term side-effects of chemotherapy for testicular cancer should be a mandatory part of any study of such treatment and should be considered in any comparison of different therapies.


Br. J. Cancer (1990), 61, 479-484 © Macmillan Press Ltd., 1990

### Long-term toxicity of chemotherapy for testicular cancer – the cost of cure

N.S.A. Stuart, C.M. Woodroffe, R. Grundy & M.H. Cullen

*Queen Elizabeth Hospital, Edgbaston, Birmingham B15 2TH, UK.*

**Summary** Twenty-seven patients cured of advanced testicular cancer by cisplatin-based chemotherapy have been assessed, a median of 30 months after start of treatment, for the long-term effects of such treatment on renal, endocrine, audiometric, reproductive and respiratory function. To control for the effects of orchidectomy on endocrine function a similar group of 11 patients cured by orchidectomy alone was also assessed. The extents of impairment in hearing and renal function were related to the total dose of cisplatin received, while the majority of patients had respiratory impairment which was, in part, related to the total dose of bleomycin. TSH was significantly higher in the chemotherapy group although serum free thyroxine and free T3 were normal in all. FSH was raised in 67% of the chemotherapy group and in 45% of the orchidectomy group while LH was raised in 75% and 45% respectively. Serum testosterone was normal in all. The levels of FSH and LH were both independently correlated with age of the patient while FSH was higher in patients having more chemotherapy and had a tendency to fall towards normal with time since treatment. Over half the patients had normal sperm concentrations although 74% had a raised proportion of abnormal sperm. Indices of sperm function were worse in patients having more chemotherapy but sperm number increased towards normal with time since treatment, particularly after the second year. The long-term side-effects of chemotherapy for testicular cancer are thus generally mild but are largely irreversible and their severity is related to the total amount of chemotherapy received. As their longer term significance is not clear we would recommend that, in the treatment of testicular cancer, doses of chemotherapy are reduced to the minimum required for cure. Assessment of long-term side-effects of chemotherapy for testicular cancer should be a mandatory part of any study of such treatment and should be considered in any comparison of different therapies.

Testicular cancer is increasing in frequency and is now the most common registered malignancy in the age group 24–34 (Davies, 1981). Cisplatin-containing chemotherapy can, however, cure the majority of patients with advanced disease (Einhorn & Williams, 1980; Newlands *et al.*, 1980). Although the immediate toxicity of such chemotherapy is well known, its long-term effects may have more impact on the quality of life of the increasing number of young men now being cured. We have studied a group of patients who have received chemotherapy for testicular cancer, with the aim of assessing its long-term effect on renal, pulmonary, endocrine, audiometric and reproductive function.

### Patients and methods

### *Patient group*

All patients of MHC and of Dr A.J. Banks who had received chemotherapy for testicular cancer in the period 1979–1983 and who remained well with no sign of recurrent disease were contacted by post and asked to take part in the study; 24 of 36 agreed. A further three had had post-chemotherapy semenalyses and were only included in this part of the analysis. One patient had a retroperitoneal teratoma and all other patients had had an orchidectomy, and although no pre-treatment semenalyses were done 10 had previously been fertile as indicated by pregnancy in their partners. No patient had hydronephrosis at presentation, six had non-bulky lung metastases and one patient had retroperitoneal lymph node dissection (RPLND) following chemotherapy. No patient had received gentamicin during therapy.

In order to assess the effect of orchidectomy alone on endocrine function a second group of 11 patients with stage I teratoma, who were free of disease a median of 11 months following orchidectomy, were selected from the patient list of the same two consultants. These had serum free thyroxine, free T3, TSH, LH, FSH and testosterone measured. The characteristics of the study groups are shown in Table I.

Correspondence: N.S.A. Stuart.
Received 9 March 1989; and in revised form 30 October 1989.

### *Methods*

Clinical notes were reviewed to obtain details of investigations at presentation and of chemotherapy received. Hepatic and renal function, serum TSH, free thyroxine and free T3, serum FSH, LH and testosterone, tumour markers ($\alpha$-fetoprotein and $\beta$-human chorionic gonadotrophin) and full blood count were measured. Standard laboratory normal ranges were used and serum hormones were measured by specific immunometric methods.

Pulmonary function assessment comprised measurement of vital capacity (VC), forced residual capacity (FRC), residual volume (RV), total lung capacity (TLC), forced expiratory capacity in one second ($FEV_1$), peak expiratory flow rate (PFR), transfer factor coefficient (KCO), total lung transfer factor (TLCO), total alveolar volume (TAV) and effective alveolar volume (EAV). TLCO was measured by single breath-hold method with correction for haemoglobin concentration, EAV by single breath helium dilution and lung volumes by steady state helium dilution. Values other than TAV and EAV were expressed as standardised residuals (Miller & Pincock, 1988) with expected values determined from height and age by published regression equations (European Coal and Steel Community Recommendations, 1983). TAV and EAV have no expected value and were expressed as absolute values.

Patients who had not had a vasectomy or RPLND (23/27) were asked to have two semenalyses. In total 36 semenalyses were done in 23 patients, 10 refusing second analysis. A median of 46 weeks (range 22–57 weeks) elapsed between semenalyses. Analyses were carried out after a mean of 4 days abstinence (range 2–14 days) with specimens produced at the laboratory and analysed immediately. The following indices were assessed according to standard criteria (WHO, 1987); volume of ejaculate, sperm number per ml, qualitative motility, per cent of motile sperm, per cent live sperm and per cent normal sperm. Mean values were used for patients having two semenalyses. Qualitative motility was determined subjectively by a trained observer and expressed as good, medium or poor.

Audiometric assessment was undertaken in a sound treated acoustic room with pure tone air conduction threshold determined for each ear at 0.5, 1, 2, 4, 6, and 8 kHz. Extent of

**Table I** Characteristics of study groups

| | *Chemotherapy group* | *Orchidectomy group* |
|---|---|---|
| Median age at diagnosis | 30 (20–51) | 26 (20–51) |
| Median months between start of treatment and time of test[a] | 30 ( 7–63) | 11 ( 1–47) |
| Median number of courses of chemotherapy (range) | 3 ( 2–11) | – |
| Mean total dose $m^{-2}$ (range) | | |
| Cisplatin | 350 mg (150–680) | – |
| Bleomycin | 168 mg ( 56–386) | – |
| Vinblastine | 41 mg ( 20– 80) | – |
| Drug regimens | | |
| Cisplatin, vinblastine, bleomycin (PVB) | 18 | – |
| PB + etoposide | 3 | – |
| PVB + etoposide | 3 | – |
| PVB + actinomycin-D | 2 | – |
| PVB + etoposide + actinomycin-D | 1 | – |
| Drug schedules[b] | | |
| Cisplatin by one hour infusion × 1 day | 18 | |
| Cisplatin by one hour infusion × 5 day | 6 | |
| Saline diuresis | 24 | |
| Additional mannitol diuresis | 6 | |
| Bleomycin bolus (i.v. or i.m.) day 1, 8, 15 | 21 | |
| Bleomycin infusion day 1–5 | 3 | |
| Mean change in weight since start of treatment (range)[b] | + 4.5kg (–4 to + 18 kg) | |

[a]Date of treatment = date of orchidectomy or start of chemotherapy; [b]excluding 3 patients who only had semen analysis.

high-tone hearing loss was defined as the total hearing loss at 6 and 8 kHz averaged for the two ears.

### *Statistical methods*

Statistical analysis was undertaken using the StatView 512+ microcomputer program (BrainPower Inc., 1986) with logistic regression carried out using the BMDP suite of software (BMDP Statistical Software, 1985). The relationship between variables was assessed by linear regression to determine the value of $b$ (slope) and of $R^2$ or by Mann-Whitney U test for the grouped variables, e.g. presence/absence of lung metastases. Forward, stepwise, multiple regression analysis was used where multiple significant associations were found with logistic regression used when the dependent variable was discontinuous (qualitative motility). The robustness of the formula derived from forward, stepwise regression was tested by backward elimination and by fitting all variables in the model. Dependent variables used were doses of cytotoxic drugs, number of courses of chemotherapy, method of chemotherapy administration (infusion versus bolus), age of patient, time since treatment and, for renal function, weight change since treatment, for pulmonary function, whether the patient was a smoker or had lung metastases and, for fertility, whether the patient had bulky abdominal disease at presentation.

### Results

### *Full blood count and biochemical profile*

All patients had normal haemoglobin, white cell and platelet count and red cell indices and had normal bilirubin, liver enzymes and tumour markers.

### *Audiometry*

There was a significant association between the extent of high-tone hearing loss and the total dose of cisplatin received per $m^2$ (Figure 1). Despite this significant association the correlation was modest and some patients had marked hearing loss at relatively low cumulative doses of cisplatin while others had little hearing loss after high doses. No association was seen between high-tone hearing loss and age at treatment, time since treatment, dose of cisplatin per course, method of cisplatin administration or type of diuresis used. Multivariate analysis confirmed that cumulative dose of cisplatin was the most highly correlated variable but also showed that, after allowing for platinum dose, patients receiving mannitol diuresis had less high-tone hearing loss (entered at step 2, $f$-to-enter 5.5, $P<0.001$).

**Figure 1** Association between total dose of cisplatin and extent of high-tone hearing loss expressed as total hearing loss at 6 kHz and 8 kHz averaged for both ears ($b = 0.008$, $R^2 = 0.2$, $P<0.03$). Patients who received additional mannitol diuresis shown by O.

### *Pulmonary function*

All patients had normal PA chest radiograph. KCO was below expected in all patients with a mean standardised residual of −1.7 (range −0.37 to −3.37). Eleven (46%) of the study group were below the lower 90% confidence interval of the expected value. KCO, however, was not related to the total dose of bleomycin received. VC and EAV each showed significant inverse associations with number of courses of chemotherapy received and with cumulative dose of bleomycin ($R^2>0.22$, $P<0.02$ in each case). These relationships are shown in Figures 2 and 3 respectively, which also indicate which patients smoked prior to treatment. Levels of VC and EAV were also lower in patients having lung metastases ($Z=-2.2$, $P<0.05$ in each case). Multivariate regression showed that VC and EAV were most highly associated with number of courses of chemotherapy received and, after allowing for this, there was no residual association with bleomycin dose, presence of metastases, whether the patient smoked or the mode of admininstration of bleomycin. Pulmonary function was also independent of age and showed no tendency to return towards normal with time after treatment.

**Figure 2** Association between total dose of bleomycin received and standardised residual of vital capacity ($b = -0.005$, $R^2 = 0.22$, $P < 0.02$). Patients who smoked before treatment shown by O.

**Figure 3** Association between total dose of bleomycin received and effective alveolar volume ($b = -0.008$, $R^2 = 0.26$, $P < 0.01$). Patients who smoked before treatment shown by O.

*Renal function*

At the start of treatment none of the 24 patients had serum urea or creatinine above the upper limit of normal (7.5 mmol l$^{-1}$ and 125 $\mu$mol l$^{-1}$, respectively) while at the time of follow-up two had serum urea and three serum creatinine levels outside this range. The majority of patients, however, showed serum creatinine and serum urea higher on follow-up than before chemotherapy (21 of 24 and 18 of 24, respectively). The extent of the change in serum creatinine between pre-treatment values and follow-up values was significantly related to the extent of treatment received (number of courses of chemotherapy or total dose of cisplatin received (Figure 4), $R^2 > 0.32$, $P < 0.005$ in each case). There was no significant association between any index of renal function and age, weight change, type of diuresis used, method of administering cisplatin or time since treatment. Multivariate analysis confirmed that cumulative dose of cisplatin was the only independently associated variable.

**Figure 4** Association between total dose of cisplatin and change in serum creatinine between pre-treatment value and post-treatment value ($b = 0.1$, $R^2 = 0.44$, $P < 0.001$).

*Endocrine function*

The results of hormone assays are summarised in Table II. Free thyroxine and free T3 levels were within the normal range in all chemotherapy and orchidectomy patients although two of 20 chemotherapy patients (10%) had raised levels of TSH. The level of thyroxine and free T3 were similar in the two groups but TSH was significantly higher in the chemotherapy group. The level of TSH was independent of age and time since treatment but was significantly associated with the cumulative dose of cisplatin and vinblastine ($R^2 = 0.13$, $P = 0.05$ in each case).

Sixteen of 24 patients in the chemotherapy group had raised LH levels (67%) while 18 (75%) had raised FSH levels. In the orchidectomy group 6/11 (55%) had raised LH and the same number raised FSH, in neither case significantly different from the chemotherapy group. All patients had testosterone levels within the normal range. Simple regression using both groups of patients showed the levels of FSH to be related to the age of the patient ($R^2 = 0.24$, $P < 0.01$), the number of courses of chemotherapy received (orchidectomy group = 0 courses, $R^2 = 0.21$, $P < 0.01$), and the cumulative doses of cisplatin, bleomycin and vinblastine (orchidectomy group = 0 mg, $R^2 > 0.11$, $P < 0.05$ in each case). Stepwise, multiple regression showed that FSH was independently associated with the number of courses of treatment ($R^2 = 0.21$, $P < 0.01$, Figure 5), the age of the patient $R^2 = 0.36$, $P < 0.01$), and inversely with time since treatment ($R^2 = 0.52$, $P < 0.001$). Levels of LH were only associated with age of the patient a finding confirmed on stepwise, multiple regression ($R^2 = 0.21$, $P < 0.01$) which showed no other independent associations.

*Semen analysis*

Table III summarises the results of semen analysis. Fifty-seven per cent of chemotherapy patients had normal sperm concentrations and, of the 19 who were not azoospermic, 17

**Table II** Serum hormone levels in chemotherapy and orchidectomy (control) groups

| *Serum hormone* | *Normal value* | *Mean (range)* | *Standard deviation* | *Number with abnormal result* |
|---|---|---|---|---|
| LH (chemotherapy group) | <8 u l$^{-1}$ | 16 (7–52) | 12 | 16/24 (67%) |
| LH (orchidectomy group) | | 10 (6–19) | 4 | 6/11 (55%) |
| FSH (chemotherapy group) | <7 u l$^{-1}$ | 16 (2–50) | 12 | 18/24 (75%) |
| FSH (orchidectomy group) | | 11 (3–22) | 7 | 6/11 (55%) |
| TSH (chemotherapy group) | <5 mu l$^{-1}$ | 2.6 (0.5–5.7) | 1.4 | 2/20 (10%) |
| TSH (orchidectomy group) | | 1.4 (0.3–4.2) | 1.0 | 0/11 |
| Free T3 (chemotherapy group) | 2.0–8.0 pmol l$^{-1}$ | 5.8 (4.2–6.9) | 0.7 | 0/20 |
| Free T3 (orchidectomy group) | | 5.4 (4.7–5.9) | 0.4 | 0/11 |
| Free thyroxine (chemotherapy group) | 90–240 nmol l$^{-1}$ | 132 (94–197) | 16 | 0/24 |
| Free thyroxine (orchidectomy group) | | 147 (125–179) | 19 | 0/11 |

For FSH, LH, Free thyroxine and free T3 no significant difference. For TSH $t = 2.4$, $P = 0.03$.

**Figure 5** Association between serum FSH and number of courses of chemotherapy received, orchidectomy group = 0 courses ($b = 2.01$, $R^2 = 0.24$, $P < 0.001$).

(89%) showed normal numbers of motile and live sperm. Three-quarters of patients, however, had a high proportion of abnormal forms. Univariate regression showed that sperm concentration was significantly associated with time since treatment (Figure 6), a finding confirmed by multivariate regression, which showed no independent association with any other variable. Indices of sperm function (per cent motile sperm, per cent live sperm, per cent normal sperm and qualitative motility) were, however, significantly associated with the amount of treatment received, i.e. number of courses of chemotherapy or cumulative dose of cisplatin or vinblastine but not with time since treatment. Stepwise, multiple regression showed that for per cent live sperm and per cent normal sperm the only independently associated variable was the cumulative dose of vinblastine received ($R^2 = 0.37$, $P < 0.01$, $R^2 = 0.23$, $P < 0.05$, respectively) while for per cent motile sperm and qualitative motility it was the cumulative dose of cisplatin ($R^2 = 0.32$, $P < 0.02$; $f$-to-enter = 20.43, $P < 0.001$, respectively).

### Discussion

For patients with incurable cancer the immediate side-effects of chemotherapy are clearly the most important and these have received most study to date. Although such side-effects

**Figure 6** Association between sperm concentration and time since start of chemotherapy, mean values plotted for patients having two semenalyses. Patients who have fathered children since chemotherapy shown by O ($b = 0.335$, $R^2 = 0.38$, $P < 0.002$).

may be severe they are essentially transient and last little longer than the treatment. For patients with curable cancer the long-term side-effects of chemotherapy may be more important as these patients have to bear any such sequelae for the rest of their lives. These long-term side-effects have received less attention.

### *Audiometry*

Previous studies have implicated a number of treatment-related factors in the development of cisplatin ototoxicity, an event which is irreversible. The duration of administration, total dose and dose per course all seem to have some influence (Piel *et al.*, 1974; Helson *et al.*, 1978; Reddel *et al.*, 1982; Schaefer *et al.*, 1985). It may also be that individual patients have different susceptibility to VIII nerve damage (Aguilar-Markulis *et al.*, 1981). Several studies have emphasised the importance of cumulative total dose, although the level above which ototoxicity was considered significant ranged from 200 to 500 mg m$^{-2}$ (Reddel *et al.*, 1982; Chiuten *et al.*, 1983; Schaefer *et al.*, 1985). This study confirms the importance of total cumulative dose with the majority of patients receiving above 300 mg m$^{-2}$ having significant hearing loss. Some patients, however, received higher cumulative doses with little hearing loss and our analysis suggests that mannitol diuresis may have some protective effect. It is possible that individual susceptibility or other factors not assessed in this study are involved in determining the extent of hearing loss. Others have shown greater ototoxicity in those aged over 40 (Melamed *et al.*, 1985), a finding that we cannot confirm because of the small number of such patients in this study.

### *Pulmonary function*

The capacity for bleomycin to cause potentially life-threatening pulmonary fibrosis is well recognised (Yagoda *et al.*, 1972). Previous studies have recommended different measures of pulmonary function for the acute assessment of bleomycin toxicity. Vital capacity, alveolar volume, pulmonary capillary blood volume and carbon monoxide diffusing capacity have all been suggested as being good measures of acute bleomycin lung damage (Van Barneveld *et al.*, 1984; Sorensen *et al.*, 1985; Luursema *et al.*, 1983). Others have found pulmonary function testing to be unhelpful (Lewis *et al.*, 1980) with no dose-related change in lung function during treatment (Bell *et al.*, 1985). Fewer studies have assessed long term pulmonary function in patients who have received bleomycin. Lucraft *et al.*, (1982) showed a dose related fall in $T_LCO$ which persisted for at least 12 months following treatment. The same study, however, showed no change in other measures of pulmonary function when the total dose of bleomycin was less than 360 mg. Luursema *et al.* (1983) also showed no change in vital capacity during treatment and up to two years afterwards. Patients in our study all had values of KCO below predicted indicating that the group as a whole had abnormal lung function following treatment. The total dose of bleomycin received was not related to the extent of impairment in KCO but was related to lung volume measurements (VC, EAV). This is consistent with, but not diagnostic of, a dose related lung fibrosis. In summary, patients treated for teratoma with chemotherapy have long term abnormalities of pulmonary function that are, at least in part, determined by the cumulative dose of

**Table III** Results of semen analysis

| *Index of fertility* | *Normal value* | *Median (range)* | *Standard deviation* | *Number with normal result* |
|---|---|---|---|---|
| Million sperm ml$^{-1}$ | >20 | 29 (0–150) | 35 | 13/23 (57%) |
| Per cent of sperm motile[a] | >50% | 58% (10–80) | 15 | 17/19 (89%) |
| Per cent of sperm alive[a] | >50% | 65% (20–86) | 15 | 16/18[b] (89%) |
| Per cent of sperm normal[a] | >45% | 38% (10–55) | 10 | 5/19 (26%) |
| Qualitative motility[a] | medium/good | – | – | 9/19 (46%) |

[a]Excluding azoospermic patients, [b]not assessed in one patient.

bleomycin and we find no evidence that these abnormalities tend to resolve with time.

*Renal function*

The tendency of cisplatin to induce renal damage is well recognised although this can be minimised by inducing a saline diuresis. In recent series the mean fall in glomerular filtration rate during cisplatin therapy has been reported as low as 0% (Swainson *et al.*, 1985) and as high as 29% (Reece *et al.*, 1987), although with different schedules of treatment. Previous studies have related the extent of cisplatin nephrotoxicity to peak levels of ultrafiltrable platinum and thus to duration of administration and dose received (Reece *et al.*, 1987). The question of cumulative toxicity is more controversial. Some have reported cumulative and unpredictable nephrotoxicity (Goren *et al.*, 1986) while others have related cumulative toxicity to renal cortical platinum concentrations (Stewart *et al.*, 1985). Others have denied any cumulative toxicity (Meijer *et al.*, 1982; Chiuten *et al.*, 1983) but have either studied small numbers of patients or have defined renal damage only when serum creatinine was above normal levels. This study has looked at long-term changes and has used each patient as his own control. We have shown that most patients have a rise in creatinine since the start of therapy and that the extent of this rise is related to the total dose of cisplatin received but is unrelated to changes in body weight. Although considerable doubt has been cast on the value of serum creatinine for monitoring renal function during chemotherapy when body mass and dietary protein intake fluctuate greatly (Daugaard *et al.*, 1988), in the stable pre-treatment or long post-treatment situation an excellent correlation between serum creatinine and GFR is seen (Daugaard *et al.*, 1988) and indeed some have suggested that serum creatinine is the preferred measure of GFR (Payne, 1986). The rise in serum creatinine in this population is thus considered to be due to renal damage which is largely determined by the cummulative dose of cisplatin. Although the extent of the damage is small, with most patients having serum urea and creatinine within the normal range, it can not be assumed to be insignificant in the long-term. This study also confirms previous findings that nephrotoxicity is irreversible and, within the age range in this study, is independent of age.

*Thyroid function*

We can find only one previous work assessing thryoid function following chemotherapy for testicular carcinoma. In this Leitner *et al.* (1986) looked at 22 patients in complete remission a median of 24 months after VAB-6 chemotherapy and showed a normal TSH in all. We confirm that the majority of patients have normal thyroid function a median of 30 months after starting chemotherapy but show that a few have raised TSH indicating increased pituitary drive and subclinical thyroid dysfunction. More interestingly we show that the popualtion who received chemotherapy had higher levels of TSH than a similar group who did not. This also suggests that there may be some subclinical thyroid damage as a result of chemotherapy and merits further investigation.

*Fertility*

Chemotherapy has historically been considered to render most patients infertile. It is now clear, however, that recovery of fertility is common following cisplatin based chemotherapy for testicular cancer (Lange *et al.*, 1983; Johnson *et al.*, 1984). This study confirms a recovery in sperm count following chemotherapy with over half the study patients having normal sperm concentration. This recovery appears most marked in the third year after treatment (Figure 6) with a majority of patients having normal sperm concentrations at this time. We have also assessed a number of sperm characterisitics which greatly influence feritility but are unable to find any similar published work. We have shown one of the determinants of the numbers of motile, normal and live sperm and of qualitative sperm motility is the extent of chemotherapy received with patients having higher cumulative doses or a greater number of courses having more abnormal values. It is also clear from this and other studies that, if the final impact of chemotherapy on fertility is to be determined, patients must be followed up for at least three years and probably longer (Kreuser *et al.*, 1986).

It has previously been reported that, following hemicastration for testicular cancer, many patients have raised LH and FSH levels (Fossa *et al.*, 1980). The majority of patients in that study, however, had also received abdominal radiation. The present study confirms raised LH and FSH in a group of patients having orchidectomy only. Patients having chemotherapy for testicular cancer commonly have raised FSH (Fossa *et al.*, 1986) although raised LH is not always seen (Kareuser *et al.*, 1986). This study shows that the proportion of patients having raised gonadotrophins is greater, and the extent of the rise more marked, in those who have also had chemotherapy. The extent of the abnormality in FSH is, in part, determined by the amount of chemotherapy received. The pattern of raised FSH following chemotherapy, falling toward normal, as seen in this study, has previously been reported (Fossa *et al.*, 1986) and parallels the recovery in sperm count that we have documented. In this study the abnormalities in LH and FSH were more marked in older patients. This may represent an effect of age alone or may be because older patients are more susceptible to the testicular effects of chemotherapy.

This study shows that most patients having cisplatin-based chemotherapy for testicular cancer have no severe long-term side-effects. The majority of patients, however, have measurable disturbance of hearing and of renal, endocrine and pulmonary function as well as of fertility. Many of these effects show an association with the amount of chemotherapy given and are thus likely to be signs of long-term dose-related toxicity. As patients with more extensive disease tend to receive more chemotherapy we cannot exclude the possibility that some of the abnormalities may be due to the extent of disease. Apart from recovery of fertility these abnormalities appear irreversible. Further follow-up is needed to assess the full significance of these abnormalities but, in the meantime, it would be appropriate, in the treatment of testicular cancer, to reduce doses of chemotherapy to the minimum required for cure. Assessment of the long-term side-effects of treatment should be a mandatory part of any study of chemotherapy for testicular cancer and such side-effects should be considered in any comparison of different therapies.

We would like to thank Dr A.J. Banks for allowing us to study his patients, Mr Brian Milton for undertaking pulmonary function testing, Jane Cuthbert, for performing semen analysis, Mr Steve Jones for clinical chemistry advice and Dr Krys Kelly for statistical advice.

**References**

AGUILAR-MARKULIS, N.V., BECKLEY, S., PRIORE, R. & METTLIN, C. (1981). Auditory toxicity effect of long term *cis*-dichlorodiammine-platinum II therapy in genitourinary cancer patients. *J. Surg. Oncol.*, **16**, 111.

BELL, M.R, MEREDITH, D.J. & GILL, P.G. (1985). Role of carbon monoxide diffusing capacity in the early detection of major bleomycin-induced pulmonary toxicity. *Aust. NZ J. Med.*, **15**, 235.

BMDP (1988). *BMDP Statistical Software*, Dixon, W.J. (ed.) University of California Press: Berkeley.

CHIUTEN, D. VOG, S., KAPLAN, B. & CAMACHO, F. (1983). Is there cumulative or delayed toxicity from cis-platinum? *Cancer*, **52**, 211.

DAUGAARD, G., ROSSING, N. & RORTH, M. (1988). Effects of cisplatin on different measures of glomerular function in the human kidney with special emphasis on high-dose. *Cancer Chemother. Pharmacol.*, **21**, 163.

DAVIES, J.M. (1981). Testicular cancer in England and Wales: some epidemiolgical aspects. *Lancet*, **i**, 928.

EINHORN, L.H. & WILLIAMS, S.D. (1980). Chemotherapy of disseminated testicular cancer. A random prospective study. *Cancer*, **45**, 1339.

EUROPEAN COAL AND STEEL COMMUNITY RECOMMENDATIONS. (1983). *Bull. Eur. Physiopathol. Respir.*, **19** (suppl. 5) , 1.

FOSSA, S.D. (1986). Fertility in patients with testicular cancer. In *Advances in the Biosciences, 55. Germ Cell Tumours II*, Jones, W.G., Milford Ward, A. & Anderson, C.K. (eds). Pergamon Press: Oxford

FOSSA, S.D., KLEEP, O & ADDKVAAG, A. (1980). Serum hormone levels in patients with malignant testicular germ cell tumours without clinical and/or radiological signs of tumour. *Br. J. Urol.*, **52**, 151.

GOREN, M.P., WRIGHT, R.K. & HOROWTIZ, M.E. (1986). Cumulative renal tubular damage associated with cisplatin nephrotoxicity. *Cancer Chemother. Pharmacol.*, **18**, 69.

HELSON, L., OKOHKWO, E., ANTON, & L. CVITKOVIC, E. (1978). Cis-platinum ototoxicity. *Clin. Toxicol.*, **13**, 469.

JOHNSON, D.H., HAINSWORTH, J.D., LINDE, R.B. & GRECO, A.F. (1984). Testicular function following combination chemotherapy with cisplatin, vinblastine and bleomycin. *Med. Pediatr. Oncol.*, **12**, 233.

KREUSER, E.D., HETZEL, H.D., HARSCH, U. & ALTWEIN, J.E. (1986). Chronic gonadal toxicity in patients with testicular cancer. In *Advances in the Biosciences, 55. Germ Cell Tumours II*, Jones, W.G., Milford Ward, A. & Anderon, C.K. (eds). Pergamon Press: London.

LANGE, P.H., NARAYAN, P., VOGELZANG, N.J., SHAFER, R.B., KENNNEDY, B.J. & FRALEY, E.E. (1983). Return of fertility after treatment for non-seminomatous testicular cancer: changing concepts. *J. Urol.*, **129**, 1131.

LEITNER, S., BOSL, G.J. & BAJORUNAS, D. (1986). Gonadal dysfuntion in patients treated for metastatic germ-cell tumours. *J. Clin. Oncol.*, **4**, 1500.

LEWIS, B.M. & IZBICKI, R. (1980). Routine pulmonary function tests during bleomycin therapy. *J. Am. Med. Assoc.*, **243**, 347.

LUCRAFT, H.H., WILKINSON, P.M., STRETTON, T.B. & READ, G. (1982). Role of pulmonary function tests in the prevention of bleomycin pulmonary toxicity during chemotherapy for metastatic testicular teratoma. *Eur. J. Cancer Clin. Oncol.*, **18**, 133.

LUURSEMA, P.B., STAR-KROESEN, M.A., VAN DER MARK, Th. W., SLEYFER, D.T. SCHRAFFORDT KOOPS, H. & PESET, R. (1983). Bleomycin-induced changes in the carbon monoxide transfer factor of the lungs and it components. *Am. Rev. Respir. Dis.*, **128**, 880.

MEIJER, S., MULDER, N.H., SLEIJFER, D. Th. & 4 others (1982). Nephrotoxicity of cis-diamminedichloro platinum (CDDP) during remission-induction and maintenance chemotherapy of testicular carcinoma. *Cancer Chemother. Pharmacol.*, **8**, 27.

MELAMED, L.B., SELIM, M.A. & SCHUCHMAN, D. (1985). Cis-platinum ototoxicity in gynaecologic cancer patients. A preliminary report. *Cancer*, **55**, 41.

MILLER, M.R. & PINCOCK, A.C. (1988). Predicted values: how should we use them. *Thorax*, **43**, 265.

NEWLANDS, E.S., BEGENT, R.H.J., KAYE, S.B., RUSTIN, G.J.S. & BAGSHAWE, K.D. (1980). Chemotherapy of advanced malignant teratoma. *Br. J. Cancer*, **42**, 378.

PAYNE, R. (1986). Creatinine clearance: a redundant clinical investigation. *Ann. Clin. Biochem.*, **23**, 243.

PIEL., MEYER, D., PERLIA, C.P. & WOLFE, V.I. (1974). Effects of diamminedichloroplatinum (NSC-119875) on hearing function in man. *Cancer Chemother. Rep.*, **58**, 871.

REDDEL, R.R., KEFFORD, R.F., GRANT, J.M., COATES, A.S., FOX, R.M. & TATTERSALL, M.N.H. (1982). Ototoxicity in patients receiving cisplatin: importance of dose and method of administration. *Cancer Treat. Rep.*, **66**, 19.

REECE, P.A., STAFFORD, I., RUSSELL, J., KHAN, M. & GILL, P.G. (1987). Creatinine clearance as a predictor of ultrafilterable platinum diposition in cancer patients treated with cisplatin: relationship between peak ultrafilterable platinum plasma levels and nephrotoxicity. *J. Clin. Oncol.*, **5**, 304.

SCHAEFER, S.D., POST, J.D., CLOSE, L.G. & WRIGHT, C.G. (1985). Ototoxicity of low and moderate dose cisplatin. *Cancer*, **56**, 1934.

SORENSEN, P.G., ROSSING, N. & RORTH, M. (1985). Carbon monoxide diffusing capacity: a reliable indicator of bleomycin-induced pulmonary toxicity. *Eur. J. Respir. Dis.*, **66**, 333.

STEWART, D.J., MIKHAEL, N.Z., NANJI, A.A. & 5 others (1985). Renal and hepatic concentrations of platinum: relationship to cisplatinum time, dose and nephrotoxicity. *J. Clin. Oncol.*, **3**, 1251.

SWAINSON, C.P., COLLS, B.M. & FITZHARRIS, B.M. (1985). Cis-platinum and distal renal tubule toxicity. *NZ Med. J.*, **98**, 375.

VAN BARNEVELD, P.W., VAN DER MARK, T.W., SLEIJFER, D.Th. & 4 others (1984). Predictive factors for bleomycin-induced pneumonitis. *Am. Rev. Respir. Dis.*, **130**, 1078.

WHO (1987). *Laboratory Manual for Examination of Human Semen and Semen-cervical Mucus Interaction*. Cambridge University Press: Cambridge.

YAGODA, A., MUKHERJI, B., YOUNG, C. & 5 others (1972). Bleomycin, an antitumour antibiotic. *Ann. Intern. Med.*, **77**, 861.